# Supplementary material for: Structural basis for APE1 processing DNA damage in the nucleosome
Source: Nat Commun. 2022 Sep 14;13:5390. doi: 10.1038/s41467-022-33057-7 (PMC9474862; doi:10.1038/s41467-022-33057-7)
Supplement: Supplementary file 1 — Supplementary Information [file 41467_2022_33057_MOESM1_ESM.pdf]

a

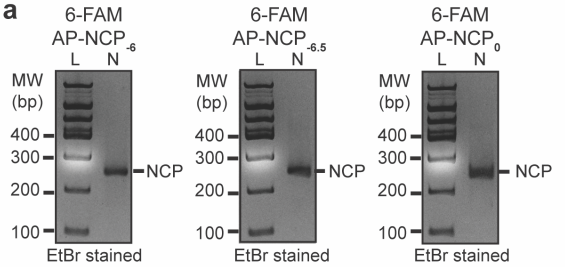

b

Substrate	k_{obs} (% major)	k_{obs} (% minor)
AP-DNA	441 ± 40 (NR)	23 ± 8 (NR)
AP-NCP$_{-6}$	500 ± 40 (84%)	0.51 ± 0.14 (16%)
AP-NCP$_{-6.5}$	0.12 ± 0.01 (67%)	$5.40x10^{-3} ± 0.51x10^{-3}$ (33%)
AP-NCP$_0$	$1.30x10^{-4} ± 0.23x10^{-4}$ (52%)	$4.50x10^{-2} ± 0.70x10^{-2}$ (48%)

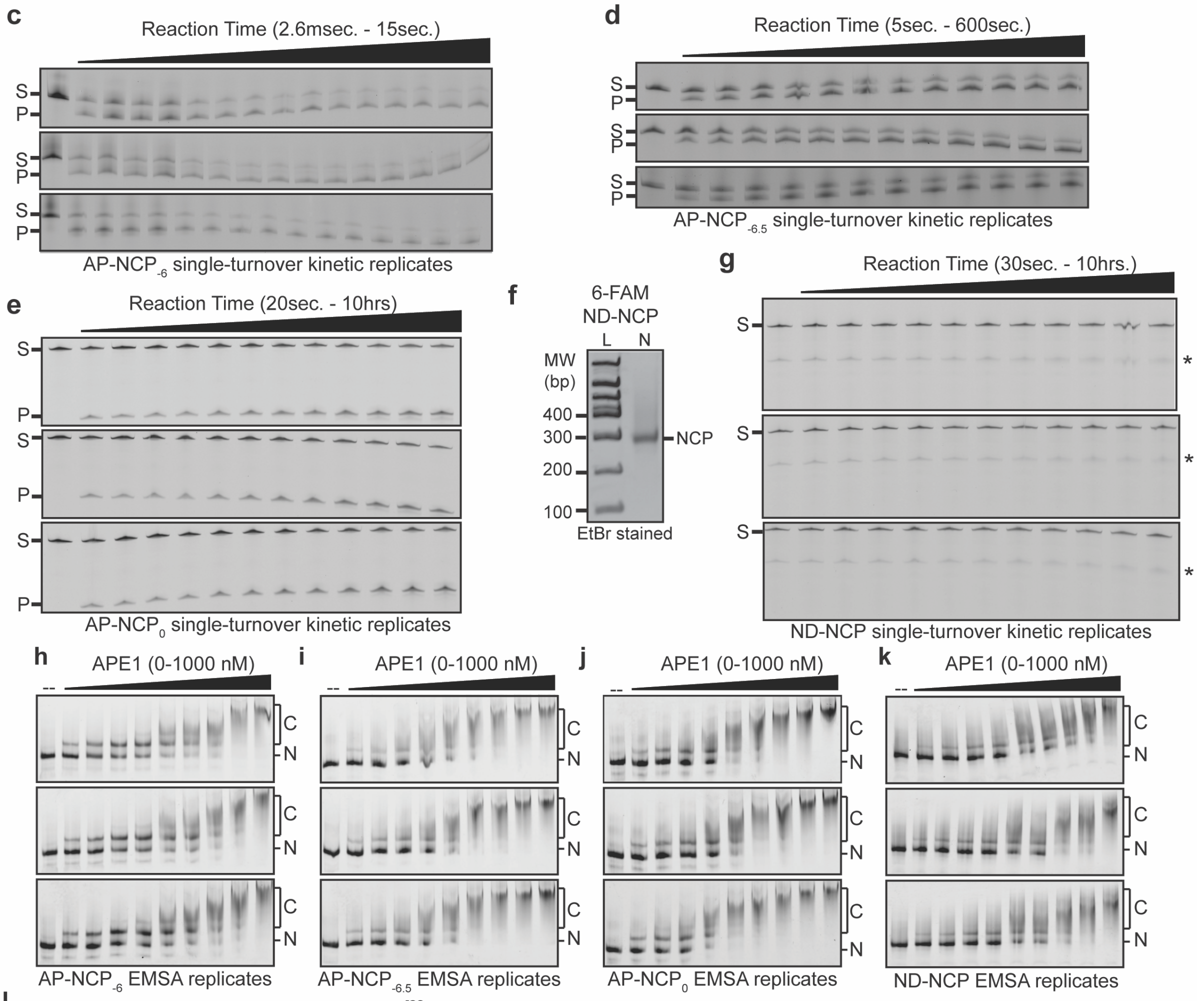

l

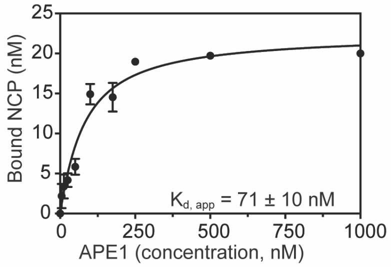

m

Substrate	K_d (nM)
AP-NCP$_{-6}$	17 ± 3
AP-NCP$_{-6.5}$	20 ± 7
AP-NCP$_{-6.5}$	20 ± 8
ND-NCP	71 ± 10

Supplementary Fig. 1. Analysis of APE1 nucleosome binding and cleavage.

a, Native PAGE gels confirming nucleosome formation for 6-FAM labeled AP-NCP$_{-6}$, AP-NCP$_{-6.5}$, and AP-NCP$_{0}$. The AP-NCPs were detected using ethidium bromide staining. The 100 bp DNA ladder (L) and nucleosome sample (S) are labeled. The Native PAGE was run after initial nucleosome generation (n=1) and run again for any experiments that occurred after one month of storage. **b,** APE1 single turnover kinetic parameters for AP-NCP$_{-6}$, AP-NCP$_{-6.5}$, and AP-NCP$_{0}$. The kinetic parameters for AP-DNA were previously reported[1]. NR stands for not reported. **c**, Gels from three replicate APE1 single turnover kinetic experiments for AP-NCP$_{-6}$. **d**, Gels from three replicate APE1 single turnover kinetic experiments for AP-NCP$_{-6.5}$. **e,** Gels from three replicate APE1 single turnover kinetic experiments AP-NCP$_{0}$. The substrate (S) and product (P) bands were detected using the 6-FAM label for (c-e). The first gel of the triplicate experiments for each AP-NCP was used as the representative gel in Fig. 1c. **f,** Native PAGE gels of 6-FAM labeled ND-NCP. The ND-NCP was detected using ethidium bromide staining. The 100 bp DNA ladder (L) and nucleosome sample (S) are labeled. The Native PAGE was run after initial nucleosome generation (n=1). **g,** APE1 single turnover pre-steady state kinetic experiments for ND-NCPs. The substrate (S) band was detected using the 6-FAM label. The asterisk represents a minor contaminant observed in the presence and absence of APE1. **h,** Gels from three replicate APE1 EMSAs for AP-NCP$_{-6}$. **i,** Gels from three replicate APE1 EMSAs for AP-NCP$_{-6.5}$. **j,** Gels from three replicate APE1 EMSAs for AP-NCP$_{0}$. **k,** Gels from three replicate APE1 EMSAs for ND-NCP. The free nucleosome (N) and complex (C) were detected using the 6-FAM label on each NCP for (h-k). **l,** Quantification of the ND-NCP EMSAs. The data shown is the mean ± standard deviation from the three replicate experiments. **m,** Table summarizing the $K_{d,app}$ calculated from the EMSAs for AP-NCP$_{-6}$, AP-NCP$_{-6.5}$, AP-NCP$_{0}$, and ND-NCP.

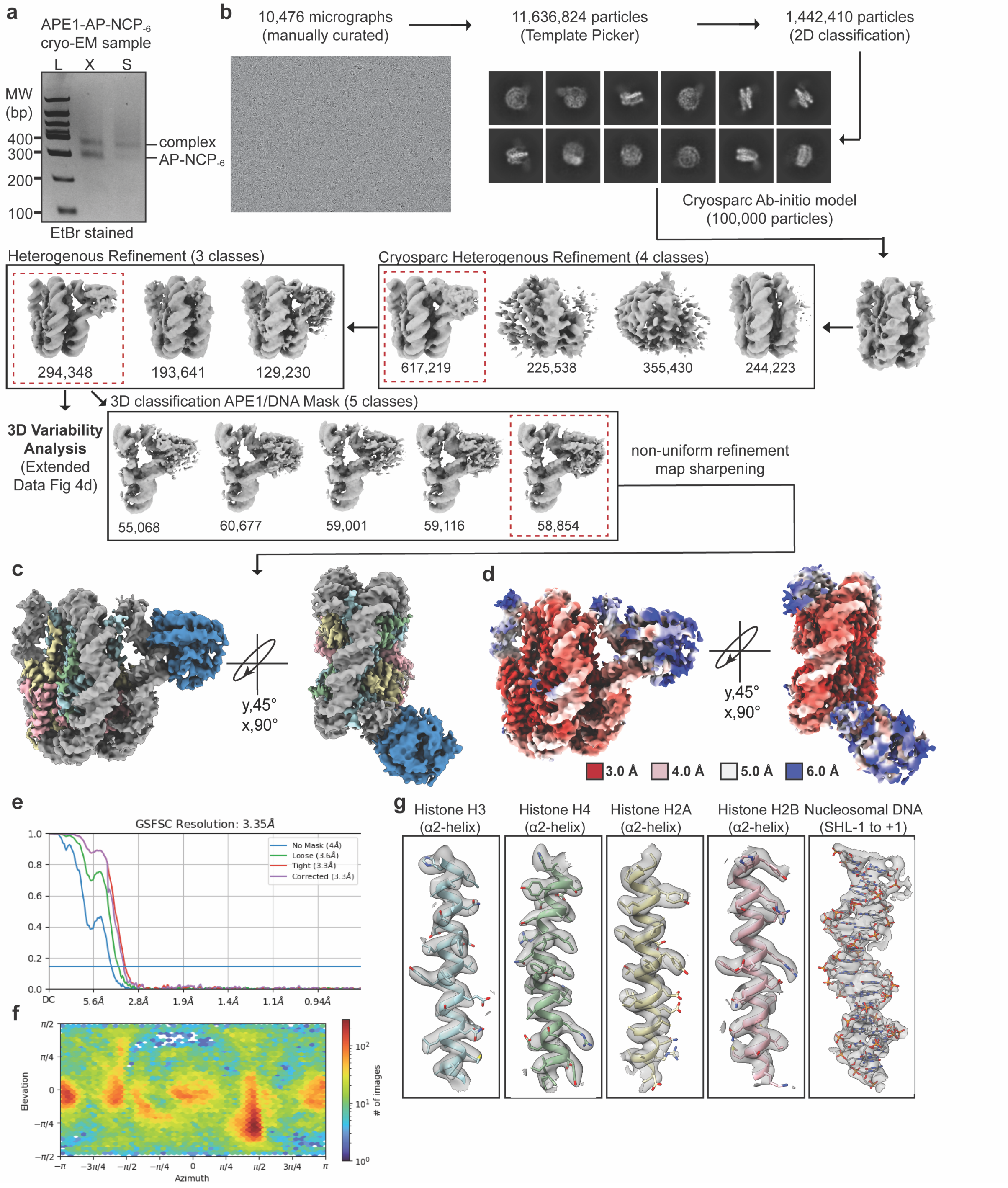
a
APE1-AP-NCP-6
cryo-EM sample
L X S
MW
(bp)
400
300
200
100
complex
AP-NCP-6
EtBr stained
b
10,476 micrographs
(manually curated)
11,636,824 particles
(Template Picker)
1,442,410 particles
(2D classification)
Cryosparc Ab-initio model
(100,000 particles)
Cryosparc Heterogenous Refinement (4 classes)
617,219
225,538
355,430
244,223
Heterogenous Refinement (3 classes)
294,348
193,641
129,230
3D classification APE1/DNA Mask (5 classes)
3D Variability
Analysis
(Extended
Data Fig 4d)
55,068
60,677
59,001
59,116
58,854
non-uniform refinement
map sharpening
c
y,45°
x,90°
d
y,45°
x,90°
3.0 Å
4.0 Å
5.0 Å
6.0 Å
e
GSFSC Resolution: 3.35Å
No Mask (4Å)
Loose (3.6Å)
Tight (3.3Å)
Corrected (3.3Å)
DC
5.6Å
2.8Å
1.9Å
1.4Å
1.1Å
0.94Å
f
Elevation
Azimuth
# of images
g
Histone H3
(α2-helix)
Histone H4
(α2-helix)
Histone H2A
(α2-helix)
Histone H2B
(α2-helix)
Nucleosomal DNA
(SHL-1 to +1)

Supplementary Fig. 2. Single particle analysis of the APE1-AP-NCP$_{-6}$ complex.

a, Native PAGE gel of the APE1-AP-NCP$_{-6}$ cryo-EM sample (n=1). The AP-NCP$_{-6}$ and APE1-AP-NCP$_{-6}$ complex bands were detected using ethidium bromide staining. The 100 bp DNA ladder (L), a sample used to generate cryo-EM grids (S), and a sample that was not used for cryo-EM grid generation (X) are labeled. **b,** For single particle analysis, micrographs were manually curated and multiple rounds of 2D classification were performed yielding a final stack of 1,442,410 particles. A representative micrograph (n=10,476) and set of 2D-classes are shown. An ab-initio model from 100,000 particles was generated prior to two rounds of heterogenous refinement (4 and 3 classes). A map containing APE1-AP-NCP$_{-6}$ was further subjected to 3D classification using a mask for APE1 and the nucleosomal DNA between SHL$_{-5.5}$ to SHL$_{-6.5}$. All maps chosen for downstream analysis are labeled by a dotted red box. **c,** Final 3.4 Å sharpened cryo-EM map of the APE1-AP-NCP$_{-6}$ complex. **d,** Local resolution estimate for the APE1-AP-NCP$_{-6}$ cryo-EM map. **e,** Fourier shell correlation (FSC-0.143) for the AP-NCP$_{-6}$ map. **f,** Heatmap of the angular distribution of particles used to generate the final APE1-AP-NCP$_{-6}$ cryo-EM map. **g,** Representative segmented density for H2A, H2B, H3, H4 and the nucleosomal DNA from the APE1-AP-NCP$_{-6}$ cryo-EM map.

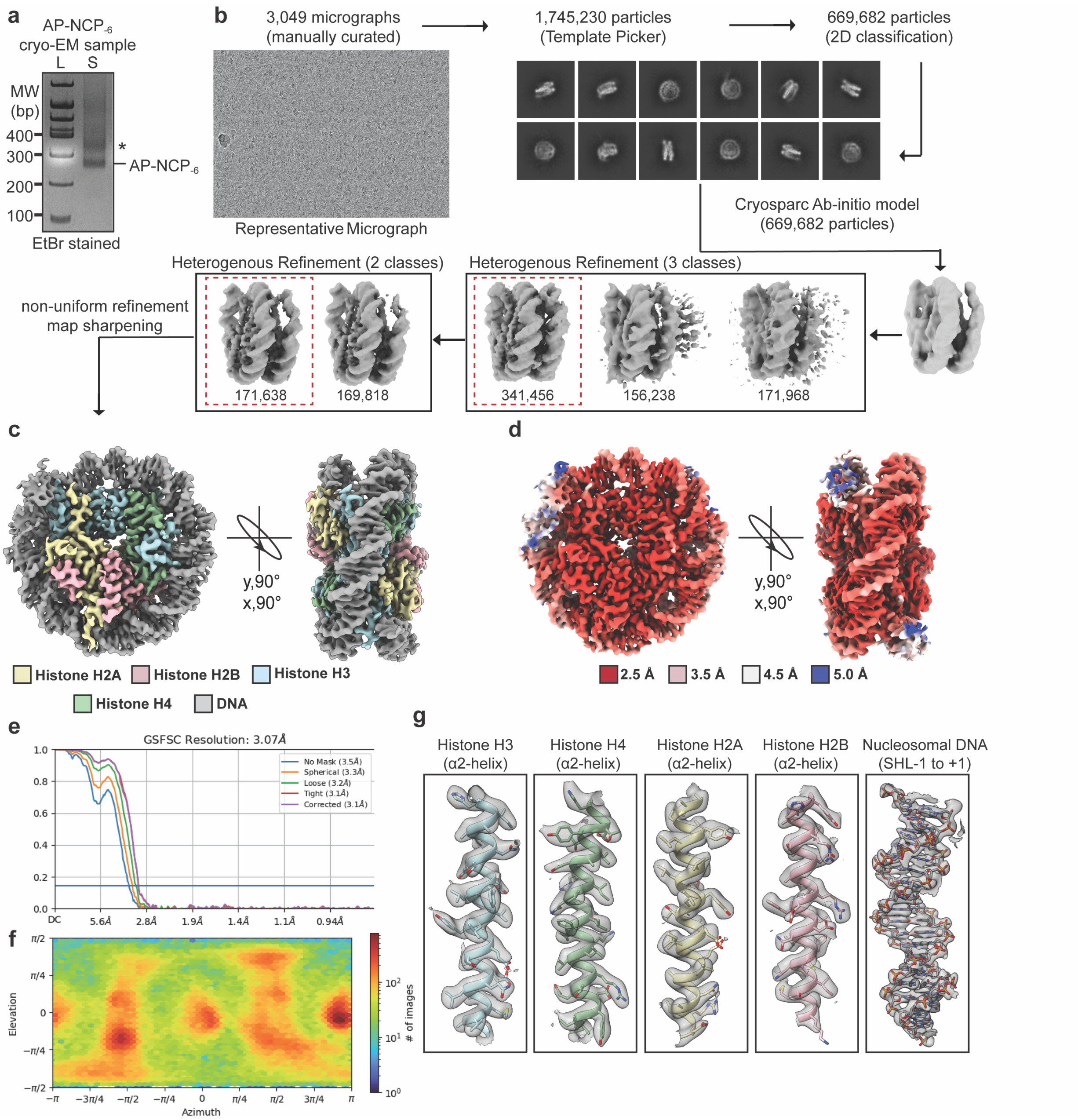

a
AP-NCP-6
cryo-EM sample
L S
MW (bp)
400
300
200
100
*
AP-NCP-6
EtBr stained
b
3,049 micrographs (manually curated)
Representative Micrograph
1,745,230 particles (Template Picker)
669,682 particles (2D classification)
Cryosparc Ab-initio model (669,682 particles)
Heterogenous Refinement (3 classes)
341,456
156,238
171,968
Heterogenous Refinement (2 classes)
171,638
169,818
non-uniform refinement
map sharpening
c
y,90°
x,90°
Histone H2A
Histone H2B
Histone H3
Histone H4
DNA
d
y,90°
x,90°
2.5 Å
3.5 Å
4.5 Å
5.0 Å
e
GSFSC Resolution: 3.07Å
No Mask (3.5Å)
Spherical (3.3Å)
Loose (3.2Å)
Tight (3.1Å)
Corrected (3.1Å)
DC
5.6Å
2.8Å
1.9Å
1.4Å
1.1Å
0.94Å
f
Elevation
Azimuth
# of images
g
Histone H3 (α2-helix)
Histone H4 (α2-helix)
Histone H2A (α2-helix)
Histone H2B (α2-helix)
Nucleosomal DNA (SHL-1 to +1)

Supplementary Fig. 3. Single particle analysis of AP-NCP$_{-6}$.

a, Native PAGE gel of the purified AP-NCP$_{-6}$ cryo-EM sample (n=1). The AP-NCP$_{-6}$ bands were detected using ethidium bromide staining. The 100 bp DNA ladder (L) and sample used to generate cryo-EM grids (S) are labeled. The asterisk represents a minor crosslinking contaminant. **b,** For single particle analysis, micrographs were manually curated and multiple rounds of 2D classification were performed yielding a final stack of 669,682 particles. A representative micrograph (n=3,049) and set of 2D-classes are shown. An ab-initio model from 669,682 particles was generated before two rounds of heterogenous refinement (3 and 2 classes). All maps chosen for downstream analysis are labeled by a dotted red box. **c,** Final 3.1 Å sharpened cryo-EM map of AP-NCP$_{-6}$. **d,** Local resolution estimate for the AP-NCP$_{-6}$ cryo-EM map. **e,** Fourier shell correlation (FSC-0.143) for the AP-NCP$_{-6}$ map. **f,** Heatmap of the angular distribution of particles used to generate the final AP-NCP$_{-6}$ cryo-EM map. **g,** Representative segmented density for H2A, H2B, H3, H4 and the nucleosomal DNA from the AP-NCP$_{-6}$ cryo-EM map.

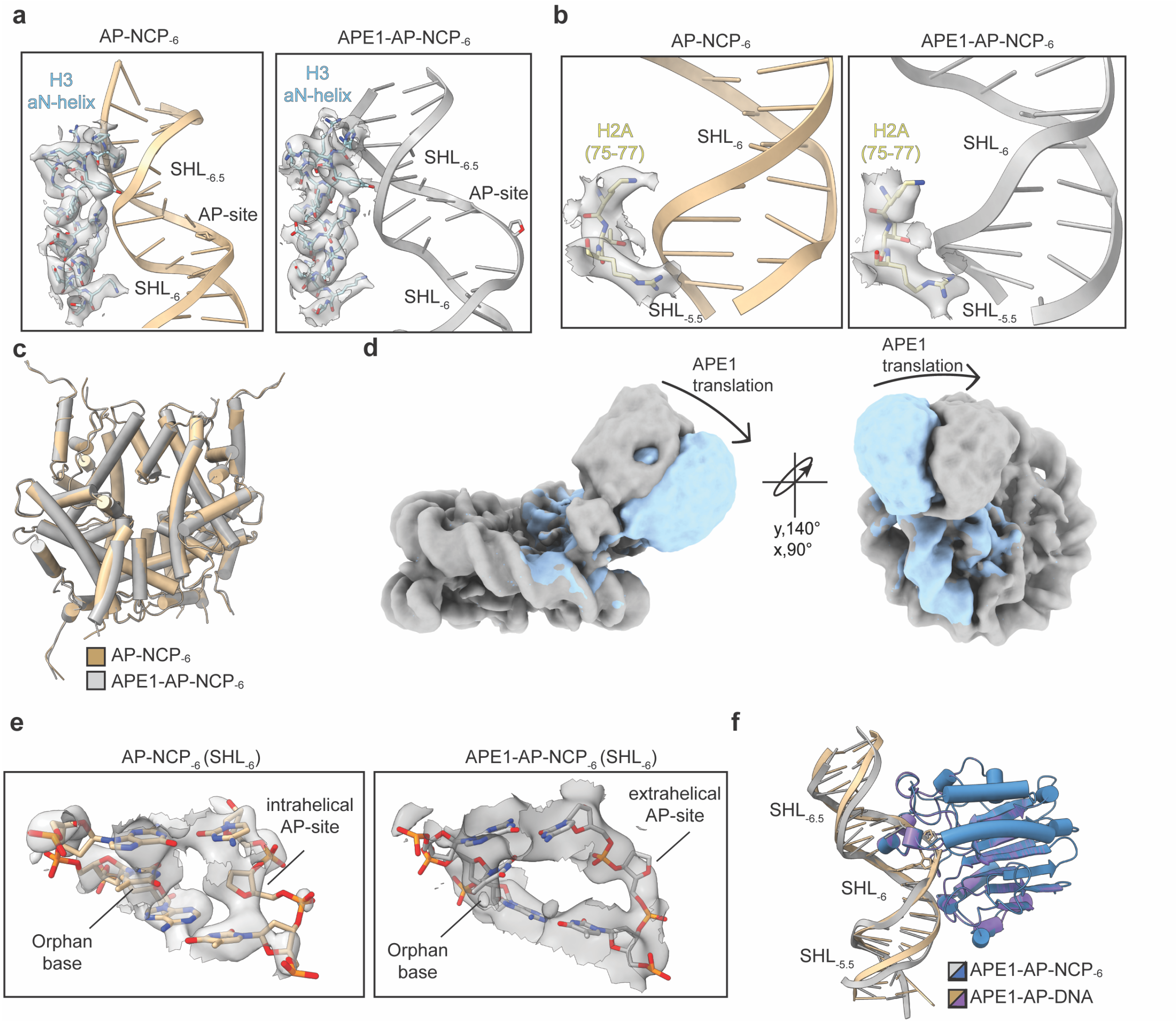
a
AP-NCP-6
APE1-AP-NCP-6
H3
aN-helix
SHL-6.5
AP-site
SHL-6
b
AP-NCP-6
APE1-AP-NCP-6
H2A
(75-77)
SHL-6
SHL-5.5
c
AP-NCP-6
APE1-AP-NCP-6
d
APE1
translation
y,140°
x,90°
e
AP-NCP-6 (SHL-6)
APE1-AP-NCP-6 (SHL-6)
intrahelical
AP-site
extrahelical
AP-site
Orphan
base
f
SHL-6.5
SHL-6
SHL-5.5
APE1-AP-NCP-6
APE1-AP-DNA

Supplementary Fig. 4. Mechanism of nucleosomal AP site recognition by APE1.

a, Focused views of the interaction between the H3 αN-helix and the nucleosomal DNA at $SHL_{-6.5}$ for $AP\text{-}NCP_{-6}$ (left) and the $APE1\text{-}AP\text{-}NCP_{-6}$ complex (right). The cryo-EM density for the H3 αN-helix is shown as a transparent gray surface. **b,** Focused views of the interactions between the H2A (residues 75-77) and the nucleosomal DNA at $SHL_{-5.5}$ for $AP\text{-}NCP_{-6}$ (left) and $APE1\text{-}AP\text{-}NCP_{-6}$ complex (right). Cryo-EM density for H2A (residues 75-77) is shown as a transparent gray surface. **c,** Structural comparison of the histone octamer from the $APE1\text{-}AP\text{-}NCP_{-6}$ and $AP\text{-}NCP_{-6}$ structures showing minimal structural rearrangements. **d,** Translational movement of APE1 around the nucleosomal DNA containing the AP site identified through 3D variability analysis. **e,** Focused views of the AP site at SHL_{-6} for $AP\text{-}NCP_{-6}$ (left) and the $APE1\text{-}AP\text{-}NCP_{-6}$ complex (right). The cryo-EM density for the nucleosomal DNA is shown as a transparent gray surface. **f,** Structural comparison of the $APE1\text{-}AP\text{-}NCP_{-6}$ and APE1-AP-DNA (PDB:5DFI) complexes.

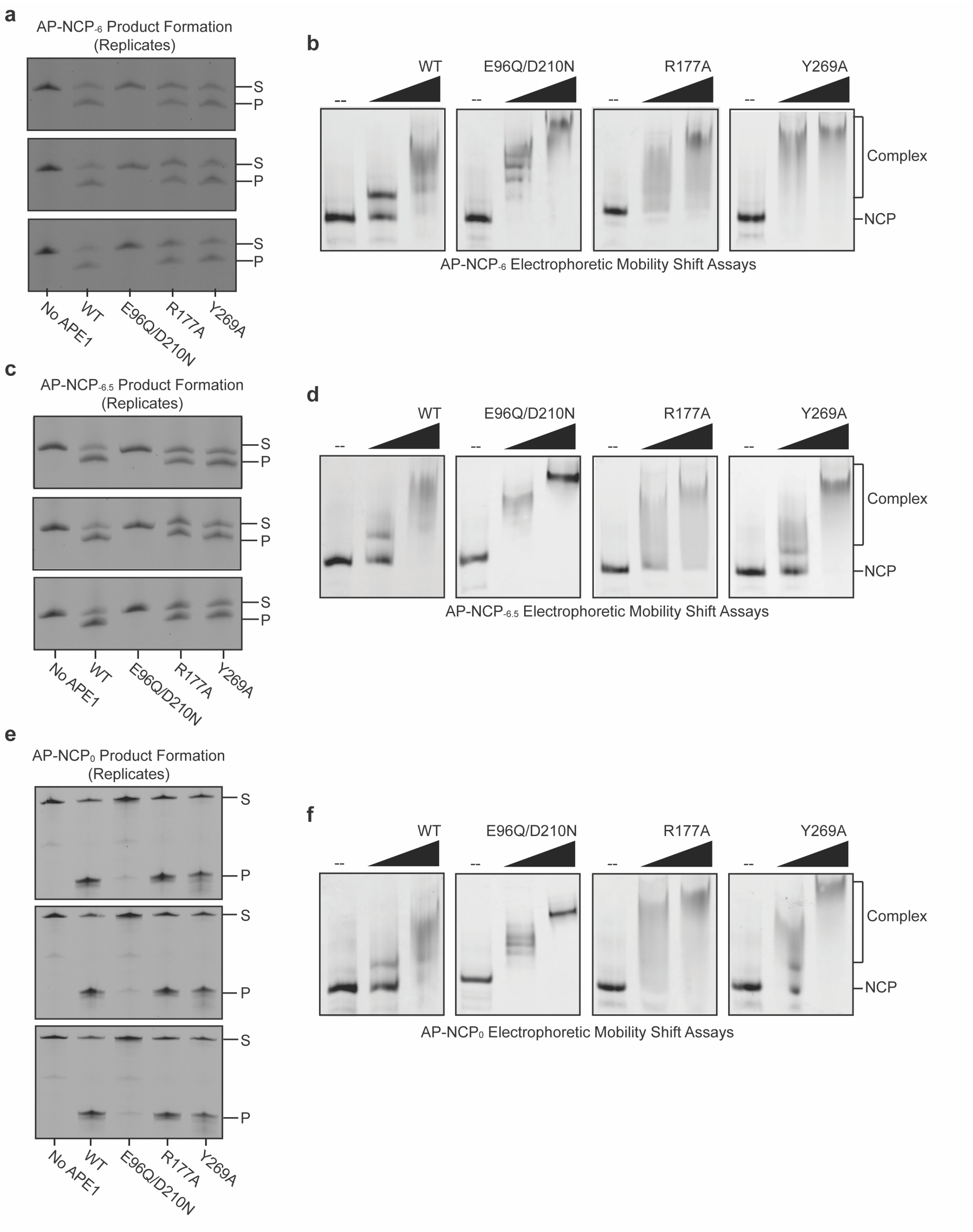
a
AP-NCP-6 Product Formation
(Replicates)
S
P
No APE1
WT
E96Q/D210N
R177A
Y269A
b
WT
E96Q/D210N
R177A
Y269A
--
Complex
NCP
AP-NCP-6 Electrophoretic Mobility Shift Assays
c
AP-NCP-6.5 Product Formation
(Replicates)
S
P
No APE1
WT
E96Q/D210N
R177A
Y269A
d
WT
E96Q/D210N
R177A
Y269A
--
Complex
NCP
AP-NCP-6.5 Electrophoretic Mobility Shift Assays
e
AP-NCP0 Product Formation
(Replicates)
S
P
No APE1
WT
E96Q/D210N
R177A
Y269A
f
WT
E96Q/D210N
R177A
Y269A
--
Complex
NCP
AP-NCP0 Electrophoretic Mobility Shift Assays

Supplementary Fig. 5. Analysis of nucleosome binding and cleavage by APE1 mutants.

a, Gels from three replicate AP-NCP$_{-6}$ product formation assays for WT, E96Q/D210N, R177A, and Y269A APE1. **b,** Representative gels of AP-NCP$_{-6}$ EMSA for WT, E96Q/D210N, R177A, and Y269A APE1 (Lanes correspond to no APE1, 25 nM APE1, and 250 nM APE1). **c,** Gels from three replicate AP-NCP$_{-6.5}$ product formation assays for WT, E96Q/D210N, R177A, and Y269A APE1. **d,** Representative gels of AP-NCP$_{-6.5}$ EMSA for WT, E96Q/D210N, R177A, and Y269A APE1 (Lanes correspond to no APE1, 25 nM APE1, and 250 nM APE1). **e,** Gels from three replicate AP-NCP$_0$ product formation assays for WT, E96Q/D210N, R177A, and Y269A APE1. **f,** Representative gels of AP-NCP$_0$ EMSA for WT, E96Q/D210N, R177A, and Y269A APE1 (Lanes correspond to no APE1, 25 nM APE1, and 250 nM APE1). The substrate (S) and product (P) bands were detected using the 6-FAM label for all product formation assays. The NCP and complex bands were detected using the 6-FAM label for all EMSAs. Source data for this figure are provided as a Source Data file.

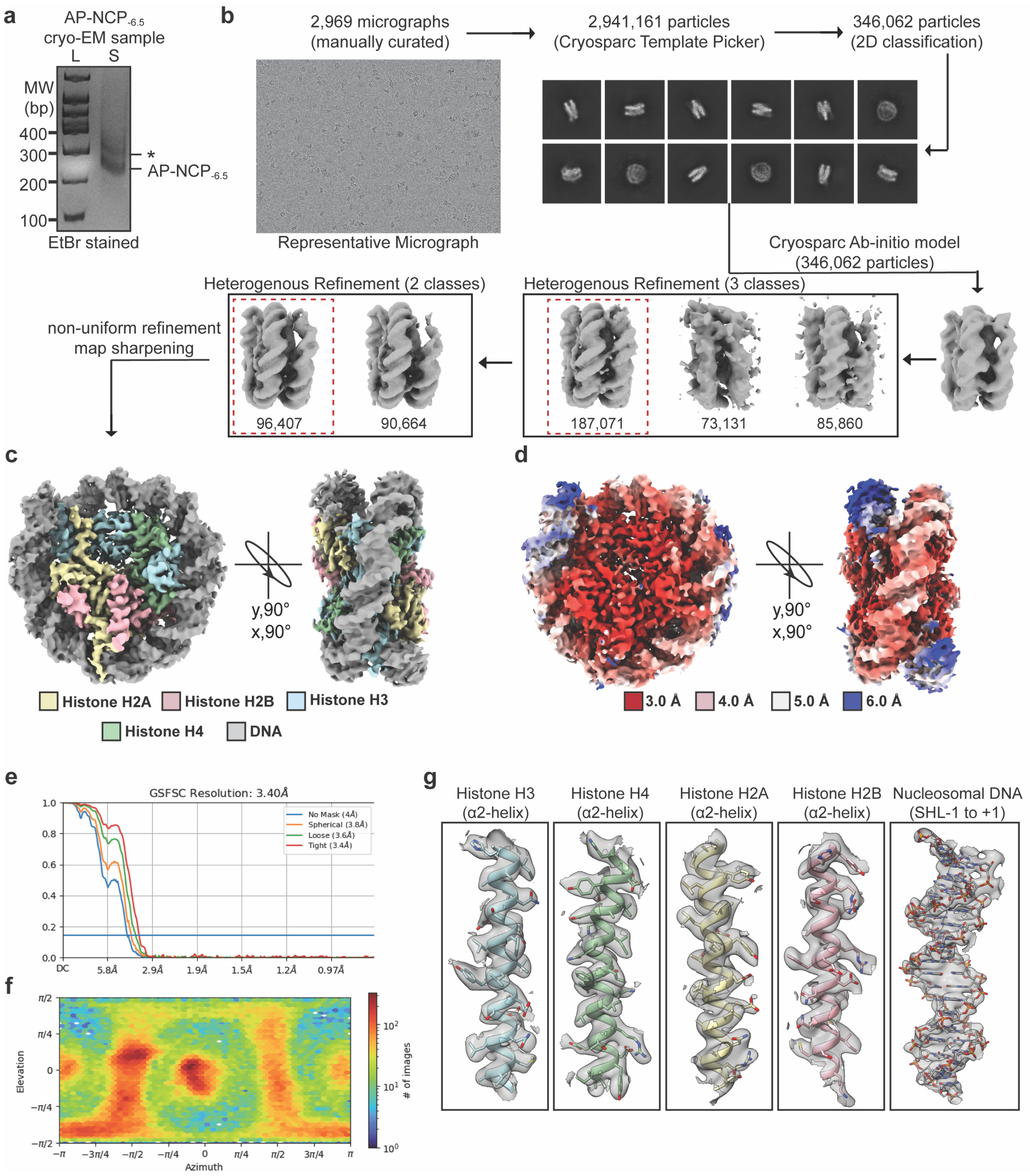

a
AP-NCP-6.5 cryo-EM sample
L S
MW (bp)
400
300
200
100
*
AP-NCP-6.5
EtBr stained
b
2,969 micrographs (manually curated)
Representative Micrograph
2,941,161 particles (Cryosparc Template Picker)
346,062 particles (2D classification)
Cryosparc Ab-initio model (346,062 particles)
Heterogenous Refinement (3 classes)
187,071
73,131
85,860
Heterogenous Refinement (2 classes)
96,407
90,664
non-uniform refinement map sharpening
c
y,90° x,90°
Histone H2A
Histone H2B
Histone H3
Histone H4
DNA
d
y,90° x,90°
3.0 Å
4.0 Å
5.0 Å
6.0 Å
e
GSFSC Resolution: 3.40Å
No Mask (4Å)
Spherical (3.8Å)
Loose (3.6Å)
Tight (3.4Å)
DC 5.8Å 2.9Å 1.9Å 1.5Å 1.2Å 0.97Å
f
Elevation
Azimuth
# of images
g
Histone H3 (α2-helix)
Histone H4 (α2-helix)
Histone H2A (α2-helix)
Histone H2B (α2-helix)
Nucleosomal DNA (SHL-1 to +1)

Supplementary Fig. 6. Single particle analysis of AP-NCP$_{-6.5}$.

a, Native PAGE gel of the purified AP-NCP$_{-6.5}$ cryo-EM sample (n=1). The AP-NCP$_{-6.5}$ bands were detected using ethidium bromide staining. The 100 bp DNA ladder (L) and sample used to generate cryo-EM grids (S) are labeled. The asterisk represents a minor crosslinking contaminant. **b,** For single particle analysis, micrographs were manually curated and multiple rounds of 2D classification were performed yielding a final stack of 346,062 particles. A representative micrograph (n=2,969) and set of 2D-classes are shown. An ab-initio model from 346,062 particles was generated before two rounds of heterogenous refinement (3 and 2 classes). All maps chosen for downstream analysis are labeled by a dotted red box. **c,** Final 3.4 Å sharpened cryo-EM map of AP-NCP$_{-6.5}$. **d,** Local resolution estimate for the AP-NCP$_{-6.5}$ cryo-EM map. **e,** Fourier shell correlation (FSC-0.143) for the AP-NCP$_{-6.5}$ map. **f,** Heatmap of the angular distribution of particles used to generate the final AP-NCP$_{-6.5}$ cryo-EM map. **g,** Representative segmented density for H2A, H2B, H3, H4 and the nucleosomal DNA from the AP-NCP$_{-6.5}$ cryo-EM map.

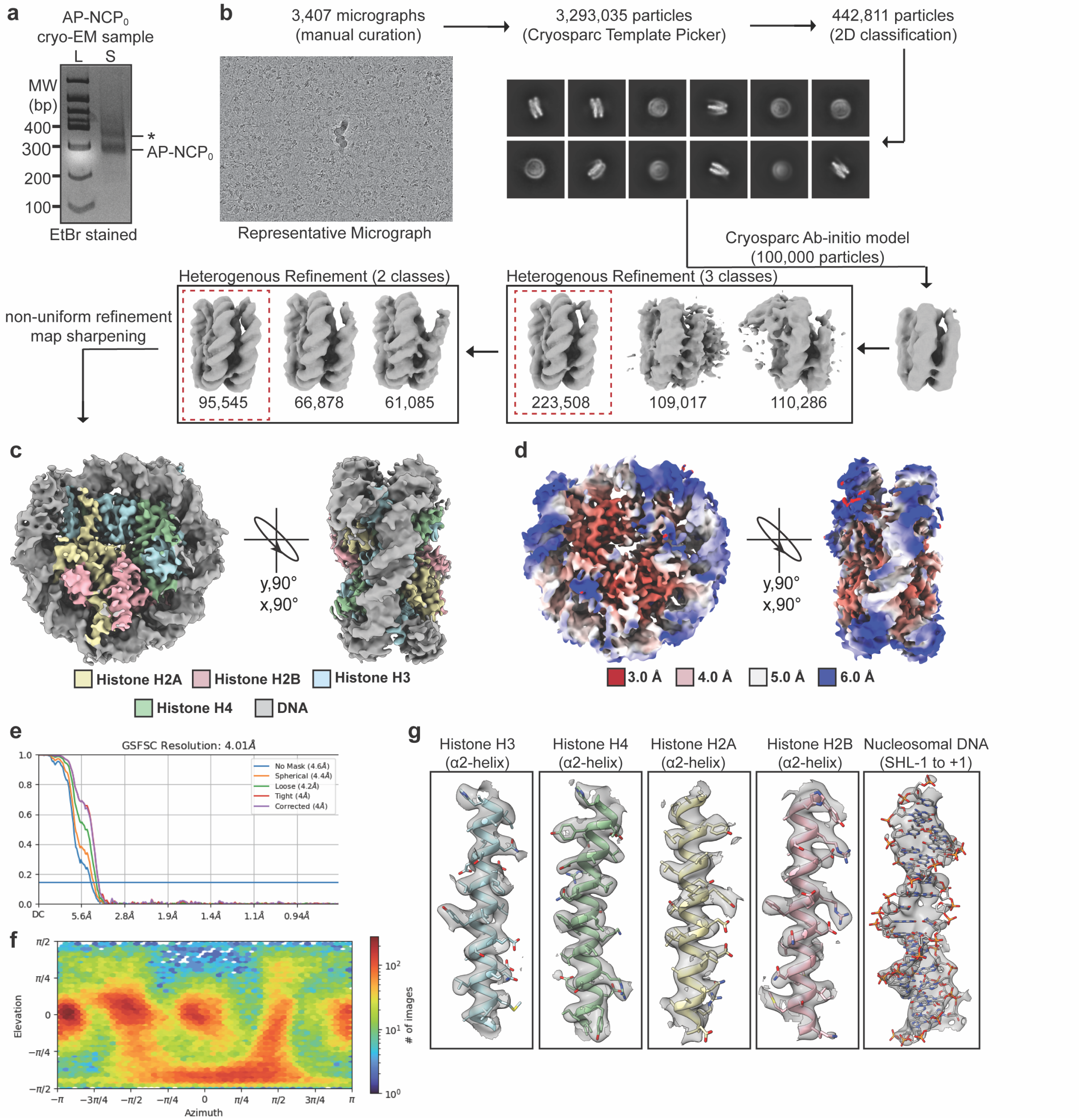
a
AP-NCP$_0$
cryo-EM sample
L S
MW
(bp)
400
300
200
100
*
AP-NCP$_0$
EtBr stained
b
3,407 micrographs
(manual curation)
3,293,035 particles
(Cryosparc Template Picker)
442,811 particles
(2D classification)
Representative Micrograph
Cryosparc Ab-initio model
(100,000 particles)
Heterogenous Refinement (3 classes)
223,508
109,017
110,286
Heterogenous Refinement (2 classes)
95,545
66,878
61,085
non-uniform refinement
map sharpening
c
y,90°
x,90°
Histone H2A
Histone H2B
Histone H3
Histone H4
DNA
d
y,90°
x,90°
3.0 Å
4.0 Å
5.0 Å
6.0 Å
e
GSFSC Resolution: 4.01Å
No Mask (4.6Å)
Spherical (4.4Å)
Loose (4.2Å)
Tight (4Å)
Corrected (4Å)
1.0
0.8
0.6
0.4
0.2
0.0
DC
5.6Å
2.8Å
1.9Å
1.4Å
1.1Å
0.94Å
f
π/2
π/4
0
−π/4
−π/2
Elevation
−π
−3π/4
−π/2
−π/4
0
π/4
π/2
3π/4
π
Azimuth
# of images
g
Histone H3
(α2-helix)
Histone H4
(α2-helix)
Histone H2A
(α2-helix)
Histone H2B
(α2-helix)
Nucleosomal DNA
(SHL-1 to +1)

Supplementary Fig. 7. Single particle analysis of AP-NCP$_0$.

a, Native PAGE gel of the purified AP-NCP$_0$ cryo-EM sample (n=1). The AP-NCP$_0$ bands were detected using ethidium bromide staining. The 100 bp DNA ladder (L) and sample used to generate cryo-EM grids (S) are labeled. The asterisk represents a minor crosslinking contaminant. **b,** For single particle analysis, micrographs were manually curated and multiple rounds of 2D classification were performed yielding a final stack of 442,811 particles. A representative micrograph (n=3,407) and set of 2D-classes are shown. An ab-initio model from 100,000 particles was generated before two rounds of heterogenous refinement (3 and 2 classes). All maps chosen for downstream analysis are labeled by a dotted red box. **c,** Final 4.0 Å sharpened cryo-EM map of AP-NCP$_0$. **d,** Local resolution estimate for the AP-NCP$_0$ cryo-EM map. **e,** Fourier shell correlation (cutoff-0.143) for the AP-NCP$_0$ map. **f,** Heatmap of the angular distribution of particles used to generate the final AP-NCP$_0$ cryo-EM map. **g,** Representative segmented density for H2A, H2B, H3, H4 and the nucleosomal DNA from the AP-NCP$_0$ cryo-EM map.

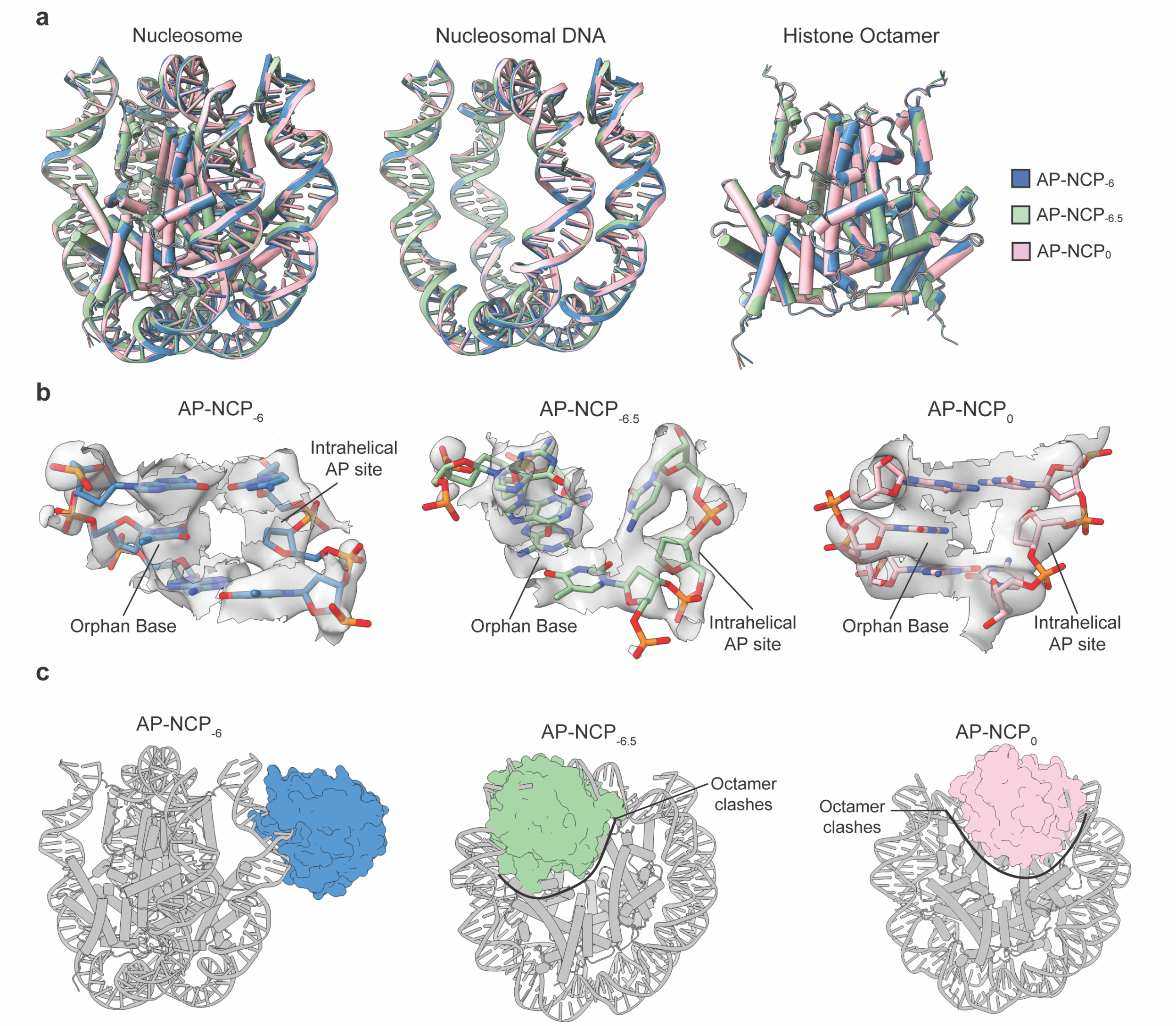
a
Nucleosome
Nucleosomal DNA
Histone Octamer
AP-NCP-6
AP-NCP-6.5
AP-NCP0
b
AP-NCP-6
Intrahelical
AP site
Orphan Base
AP-NCP-6.5
Orphan Base
Intrahelical
AP site
AP-NCP0
Orphan Base
Intrahelical
AP site
c
AP-NCP-6
AP-NCP-6.5
Octamer
clashes
AP-NCP0
Octamer
clashes

Supplementary Fig. 8. Mechanism for APE1 processing occluded AP sites in the nucleosome.

a, Overall comparison of the AP-NCP$_{-6}$, AP-NCP$_{-6.5}$, and AP-NCP$_{0}$ structures. **b,** Focused views of the intrahelical AP site for AP-NCP$_{-6}$ (left), AP-NCP$_{-6.5}$ (middle), and AP-NCP$_{0}$ (right). The cryo-EM map is shown as a transparent gray surface. **c,** Cryo-EM structure of the APE1-AP-NCP$_{-6}$ complex (left), and structural models of APE1 positioned to interact with the AP sites on AP-NCP$_{-6.5}$ (middle) and AP-NCP$_{0}$ (right). APE1 is shown as a surface representation. Significant clashes of APE1 with the histone octamer are labeled.

Supplementary Table 1. Cryo-EM data collection, refinement, and validation.

Structure	APE1-AP-NCP$_{-6}$	AP-NCP$_{-6}$	AP-NCP$_{-6.5}$	AP-NCP$_{0}$
PDB accession	7U50	7U51	7U52	7U53
EMDB accession	EMD-26336	EMD-26337	EMD-26338	EMD-26339
**Data collection and processing**				
Magnification	29,000x	29,000x	105,000x	29,000x
Voltage (kV)	300	300	300	300
Electron exposure (e^{-}/Å^{2})	50	50	47	50
Defocus range (µm)	-0.8 to -2.2	-0.8 to -2.2	-0.7 to -2.1	-0.8 to -2.0
Pixel size (Å)	0.40075	0.40075	0.415	0.40075
Symmetry imposed	C1	C1	C1	C1
Initial particle images (no.)	1,442,410	669,682	346,062	442,811
Final particle images (no.)	58,854	171,638	96,407	95,545
Map resolution (Å)	3.4	3.0	3.4	4.0
FSC threshold	0.143	0.143	0.143	0.143
**Refinement**				
Initial model used (PDB ID)	4JJN, 5DFI	4JJN	4JJN	4JJN
Model resolution (Å)	3.4	3.0	3.3	3.9
FSC threshold	0.143	0.143	0.143	0.143
**Model composition**				
Nonhydrogen atoms	14,028	11,883	11,847	11,828
Protein residues	1025	750	746	748
Nucleotide	288	290	290	288
**B factors (Å^2)**				
Protein	81	17	46	61
Nucleotide	129	78	119	130
**r.m.s. deviations**				
Bond Length (Å) (# > 4σ)	0.009 (49)	0.006 (9)	0.005 (3)	0.005 (5)
Bond Angles (°) (# > 4σ)	1.106 (95)	0.710 (8)	0.663 (6)	0.763 (24)
**Validation**				
MolProbity score	1.96	1.49	1.48	1.72
Clashscore	7.78	6.12	9.00	11.22
Poor rotamers (%)	2.45	0.80	0.48	1.29
**Ramachandran plot**				
Favored (%)	96.4	97.1	98.8	97.7
Allowed (%)	3.6	2.9	1.2	2.3
Disallowed (%)	0	0	0	0

Supplementary Table 2. Oligonucleotides for generating damaged nucleosomes.

Oligo	Sequence (5′ – 3′)
ND-NCP oligos	Single turnover enzyme kinetics, EMSAs, and product formation assays
ND_001	ATCGGATGTATATATCTGACACGTGCCTGGAGACTAGGGAGTAATCCCCTTG GCGGTTAAAACGCGGGGGACAG
ND_002	/5Phos/CGCGTACGTGCGTTTAAGCGGTGCTAGAGCTGTCTACGACCAATTGA GCGGCCTCGGCACCGGGATTCTCGAT
ND_003*	/56-FAM/ATCGAGAATCCCGGTGCCGAGGCCGCTCAATTGGTCGTAGACA GCTC
ND_004	/5Phos/TAGCACCGCTTAAACGCACGTACGCGCTGTCCCCCGCGTTTTAACCGC CA
ND_005	/5Phos/AGGGGATTACTCCCTAGTCTCCAGGCACGTGTCAGATATATACATCC GAT
AP-NCP$_{-6}$ oligos	Cryo-EM, single turnover enzyme kinetics, EMSAs, and product formation assays
AP-NCP$_{-6}$_001	ATCGGATGTATATATCTGACACGTGCCTGGAGACTAGGGAGTAATCCCCTTG GCGGTTAAAACGCGGGGGACAG
AP-NCP$_{-6}$_002	/5Phos/CGCGTACGTGCGTTTAAGCGGTGCTAGAGCTGTCTACGACCAATTGA GCGGCCTCGGCACCGGGATTCTCGAT
AP-NCP$_{-6}$_003*	/56-FAM/ATCGAGAATCCCGGTGCCGAGGCCGCTCAATTGGTCGTAGACA GCTC
AP-NCP$_{-6}$_004	/5Phos/TAGCACCGCTTAAACGCACGTACGCGCTGTCCCCCGCGTTTTAACCGC CA
AP-NCP$_{-6}$_005	/5Phos/AGGGGATTACTCCCTAGTCTCCAGGCACGTGTCAGATATAT/THF/CAT CCGAT
AP-NCP$_{-6.5}$ oligos	Cryo-EM, single turnover enzyme kinetics, EMSAs, and product formation assays
AP-NCP$_{-6.5}$_001	ATCGGATGTATATATCTGACACGTGCCTGGAGACTAGGGAGTAATCCCCTTG GCGGTTAAAACGCGGGGGACAG
AP-NCP$_{-6.5}$_002	/5Phos/CGCGTACGTGCGTTTAAGCGGTGCTAGAGCTGTCTACGACCAATTGA GCGGCCTCGGCACCGGGATTCTCGAT
AP-NCP$_{-6.5}$_003*	/56-FAM/ATCGAGAATCCCGGTGCCGAGGCCGCTCAATTGGTCGTAGACA GCTC
AP-NCP$_{-6.5}$_004	/5Phos/TAGCACCGCTTAAACGCACGTACGCGCTGTCCCCCGCGTTTTAACCGC CA
AP-NCP$_{-6.5}$_005	/5Phos/AGGGGATTACTCCCTAGTCTCCAGGCACGTGTCAGATATATA CAT/THF/CGAT
AP-NCP$_0$ oligos	Cryo-EM, single turnover enzyme kinetics, EMSAs, and product formation assays
AP-NCP$_{-6.5}$_001	ATCGGATGTATATATCTGACACGTGCCTGGAGACTAGGGAGTAATCCCCTTG GCGGTTAAAACGCGGGGGACAG
AP-NCP$_{-6.5}$_002	/5Phos/CGCGTACGTGCGTTTAAGCGGTGCTAGAGCTGTCTACGACCAATTGA GCGGCCTCGGCACCGGGATTCTCGAT
AP-NCP$_{-6.5}$_003*	/56-FAM/ATCGAGAATCCCGGTGCCGAGGCCGCTCAATTGGTCGTAGACA GCTC
AP-NCP$_{-6.5}$_004	/5Phos/TAGCACCGCTTAAACGCACGTACGC/THF/CTGTCCCCCGCGTTTTAAC CGCCA
AP-NCP$_{-6.5}$_005	/5Phos/AGGGGATTACTCCCTAGTCTCCAGGCACGTGTCAGATATATACATCC GAT

*These oligos do not contain the 6-FAM label for cryo-EM substrates.

Supplementary References

1. Maher RL, Bloom LB. Pre-steady-state kinetic characterization of the AP endonuclease activity of human AP endonuclease 1. *Journal of Biological Chemistry* **282**, 30577-30585 (2007).
